# The Functional Characterization of an AA10 Lytic Polysaccharide Monooxygenase from *Saccharophagus degradans* 2-40^T^ for Enhanced Chitin Biodegradation

**DOI:** 10.3390/foods14162839

**Published:** 2025-08-16

**Authors:** Dan Wu, Meiling Dan, Mu-Rong Kao, Yanping Li, Jiajia Song, Yuting Zheng, Guohua Zhao, Yves S. Y. Hsieh, Damao Wang

**Affiliations:** 1 College of Food Science, Southwest University, Chongqing 400715, Chinazyt20040817@email.swu.edu.cn (Y.Z.); 2 College of Food Science, Yibin Academy of Southwest University, Yibin 644000, China; 3 School of Pharmacy, College of Pharmacy, Taipei Medical University, Taipei 10031, Taiwan; murong115@tmu.edu.tw; 4 College of Life Science, Sichuan Normal University, Chengdu 610101, China; 5 Division of Glycoscience, Department of Chemistry, School of Engineering Sciences in Chemistry, Biotechnology and Health, KTH Royal Institute of Technology, AlbaNova University Centre, 106 91 Stockholm, Sweden

**Keywords:** Lytic polysaccharide monooxygenases, AA10 family, Chitin, synergistic action

## Abstract

Lytic polysaccharide monooxygenases (LPMOs) represent copper-dependent enzymes pivotal in breaking down resilient polysaccharides like cellulose and chitin by means of oxidation, creating more accessible sites for glycoside hydrolases. To elevate the conversion efficiency of chitin, an AA10 LPMO was identified from the genome of *Saccharophagus degradans* 2-40^T^ and heterologously expressed. The optimal pH for the activity of recombinant *Sd*LPMO10A is 9.0, and the optimal temperature is 60 °C. Assessment of *Sd*LPMO10A’s synergism with commercial chitinase indicated that when comparing the enzyme combination’s activity to the activity of chitinase alone, the synergistic effect was significant, and a one-pot reaction appeared superior to a two-step reaction. This discovery of a functional AA10 family LPMO presents a promising avenue for developing highly efficient catalysts for biomass conversion of chitin-rich food processing waste (e.g., shrimp shells) into bioactive chitooligosaccharides with applications in functional foods, such as prebiotics and antioxidants.

## 1. Introduction

Chitin, composed of *N*-acetyl-D-glucosamine (GlcNAc, NAG) monomers linked by β-1,4 glycosidic bonds, is the second most abundant insoluble polysaccharide [1]. Extensive research has demonstrated the diverse biological activities of *N*-acetyl chitooligosaccharides, including anti-tumor, antioxidant, anti-inflammatory, antimicrobial, immunomodulatory, and plant elicitor activities, prebiotic effects, and the ability to improve diabetes or its related complications and to reduce the development of atherosclerosis [2]. In food science and technology, these chitooligosaccharides serve as promising functional ingredients in nutraceuticals, food supplements, and fortified products, contributing to health benefits like enhanced gut microbiota modulation and reduced oxidative stress in processed foods [2]. Moreover, efficient enzymatic degradation of chitin is important for food waste management in the seafood industry, promoting circular economy principles in food production. Fully harnessing chitin resources holds immense promise in addressing shell waste pollution while amplifying the value-added utilization of by-products. The tightly packed hydrogen bond network in crystalline chitin fibers limits water molecule penetration, impeding effective degradation by glycoside hydrolases (GHs), but the discovery of lytic polysaccharide monooxygenases (LPMOs) has introduced a breakthrough in addressing this challenge. LPMOs facilitate glycosidic bond cleavage and create new chain ends by oxidizing the crystalline regions of polysaccharides, thereby producing degradation points accessible to GHs [3]. The synergistic action of LPMOs and GHs significantly enhances overall biomass degradation.

LPMOs are copper-dependent enzymes, and their active site is formed though the coordination of a copper ion to a histidine brace, which is composed of two conserved histidine residues, one of which is at the *N*-terminus [4]. As monooxygenases or true peroxygenases, LPMOs cleave polysaccharide chains by oxidizing the glycosidic bond at the C1 and/or C4 sites in the presence of electron donors and the co-substrates O_2_ or H_2_O_2_. In the monooxygenase reaction, in addition to one O_2_ molecule, two externally delivered electrons are required to complete each catalytic cycle (R-H + O_2_ + 2e^−^ + 2H^+^ → R-OH + H_2_O) [5]. One electron is used for copper reduction at the active site, which is the initial step of LPMO catalysis, while the role of the other is not yet clear. However, in the peroxygenase reaction, just a single electron is required for the “priming”, followed by the addition of H_2_O_2_ for achieving multiple catalytic cycles (R-H + H_2_O_2_ → R-OH + H_2_O) [6]. LPMOs hydroxylate the C1 carbon to form δ-1,5-lactone and the C4 carbon to generate 4-ketoaldose. Subsequently, both δ-1,5-lactone and 4-ketoaldose undergo spontaneous hydration, which leads to the formation of aldonic acid and geminal diol, respectively [6]. At present, only C1-oxidized products are observed as the result of reactions catalyzed by chitin-active LPMOs, while some cellulose-active LPMOs can also produce C4-oxidized products [7]. LPMOs encompass eight auxiliary activity (AA) families (AA9-AA11 and AA13-AA17) [8], each with distinct substrate specificity. Among these, only AA10, AA11, and AA15 LPMOs have demonstrated potential chitin cleavage activity. AA10 LPMOs, predominantly found in bacteria, exhibit cellulose and/or chitin activity, with only a handful of them concurrently acting on both substrates. Thus, exploring the application of functional enzymes within this family holds significant promise in the bio-refinery domain.

*Saccharophagus degradans* 2-40^T^, an aerobic saprophyte isolated from decaying saltwater marsh grass (*Spartina alterniflora*) in an estuary, expresses diverse enzyme systems capable of decomposing over 10 complex polysaccharides, even degrading whole plant materials [9,10]. Its genome harbors numerous gene models encoding enzymes, including glycoside hydrolase domains, glycoside transferases, polysaccharide lyases, and carbohydrate esterases [11]. In this study, a putative chitin-active LPMO was identified within the genome of *S. degradans* 2-40^T^, comprising an AA10 catalytic domain and a family 2 carbohydrate-binding module (CBM). Subsequently, *Sd*LPMO10A was cloned, expressed in *Escherichia coli*, and subjected to enzymatic characterization and mode of action analysis. This enzyme is the first LPMO to be characterized in *S. degradans* 2-40^T^. Our research will provide positive implications for the study of LPMOs in the field of chitin degradation.

## 2. Materials and Methods

### 2.1. Materials and Chemicals

α-Chitin from shrimp shells (practical grade, coarse flakes) and all the reagents used were from Sigma-Aldrich (St. Louis, MO, USA). Chitinase from *Streptomyces griseus* (200 U/g), an endo chitinase with a molecular weight of 30 kDa from the GH18 family, was purchased from Shanghai Yingxin laboratory equipment Co., Ltd. (Shanghai, China). Ezup Column Bacteria Genomic DNA Purification Kit, sodium dodecyl sulfate–polyacrylamide gel electrophoresis (SDS-PAGE) preparation kit, and BCA protein assay kit were acquired from Sangon Biotech Co., Ltd. (Shanghai, China). XhoI and NdeI restriction enzymes and T_4_ DNA ligase were provided by Thermo Fisher Scientific (Waltham, MA, USA). *E. coli* DH5α and BL21 (DE3) competent cells were from Solarbio Biotech Co., Ltd. (Beijing, China).

### 2.2. Cloning, Expression, and Purification

The *S. degradans* 2-40^T^ strain, previously preserved at −80 °C, was activated using Marine Broth 2216 (Becton, Dickinson and Company, Sparks, MD, USA) at 30 °C. Genomic DNA extraction employed the Ezup Column Bacteria Genomic DNA Purification Kit, and the DNA was stored at −20 °C. Signal peptide analysis of *Sd*LPMO10A encoded by the *Sde*_0633 gene (GenBank ID: ABD79895.1) from the *S. degradans* 2-40^T^ genome available on the National Center for Biotechnology Information (NCBI) database was conducted via SignalP-6.0, followed by primer design using Serial Cloner 2.6.1. PCR amplification, utilizing theoretical primers containing the XhoI and NdeI restriction enzyme recognition sequences (F: 5′-CGGGATCCGGCTTAATGGTAGACCCG-3′, R: 5′-CCCTCGAGATTACAAACCGCACCCGTT-3′), was performed with the *S. degradans* 2-40^T^ strain genome as a template within an applied system (SimpliAmp^TM^, Thermo Fisher Scientific, Inc., MA, USA). The resultant gene encoding *Sd*LPMO10A was directly cloned with the pelB signal peptide in the pET-26b (+) vector through ligation using T_4_ DNA ligase. Validation of the gene was performed through DNA sequencing after transformation into *E. coli* DH5α cells via heat shock treatment. Subsequently, the successfully sequenced recombinant plasmid (pET26b-*Sd*LPMO10A) was further transferred into *E. coli* BL21 (DE3) cells for protein expression.

The protein expression process involved culturing the cells in 20 mL of Luria–Bertani (LB) medium supplemented with 30 μg/mL of kanamycin at 37 °C under shaking at 200 rpm until the culture’s OD_600_ reached 0.6–0.8. Subsequently, a 2‰ seed volume was inoculated into additional LB medium containing 30 μg/mL of kanamycin and incubated until the OD_600_ reached 0.8–1.0. Induction of protein expression occurred by adding isopropyl β-D-1-thiogalactopyranoside (IPTG) at a final concentration of 1 mM, followed by overnight incubation at 30 °C and 180 rpm.

Protein extraction was conducted using a modified cold osmotic shock method based on Wang et al. [12]. The cells were centrifuged at 10,000× *g* at 4 °C for 5 min and resuspended in ice-cold buffer A, which contained 0.2 M Tris-HCl, pH 8.0, 200 g/L of sucrose, and 0.1 M EDTA-2Na. Then the lysate was incubated on ice for 20 min and centrifuged at 10,000× *g* for 15 min to collect the precipitate. The subsequent steps involved resuspension in ice-cold buffer B, which included 0.01 M Tris-HCl, pH 8.0, 0.005 M MgSO_4_, 0.002 M SDS, and 1% (*v*/*v*) Triton X-100, followed by incubation on ice for 20 min and centrifugation at 10,000× *g* for 40 min to collect the supernatant. The supernatant, containing periplasmic and membrane proteins, was filtered using a 0.45 μm membrane (Tianjin Jinteng Experiment Equipment Co., Ltd., Tianjin, China).

Purification of the recombinant *Sd*LPMO10A protein was achieved through a HisSep Ni-NTA 6FF chromatography column (Shanghai Yeasen Biotechnology Co., Ltd., Shanghai, China). Elution of the target proteins from the column was performed with elution buffers containing 50–100 mM imidazole after column equilibration with wash buffer, including 20 mM imidazole. Assessment of the recombinant protein’s purity was conducted via SDS-PAGE (Bio-Rad Laboratories, Shanghai, China). The concentration of the purified *Sd*LPMO10A protein was determined using the BCA protein assay kit, following concentration using an Amicon ultracentrifuge filter unit with a nominal molecular weight cut-off of 10,000 Da (Millipore, Cork, Ireland). The Cu^2+^-saturated form of *Sd*LPMO10A was acquired by incubating with an equimolar solution of CuCl_2_ at 30 °C for 1 h for subsequent use.

### 2.3. Phylogenetic Analysis and Structural Sequence Alignment

The N-terminal signal peptide and any C-terminal CBM domains of AA10 LPMOs were manually removed, and only the catalytic domains were preserved for phylogenetic analysis and multiple sequence alignment. A maximum-likelihood phylogenetic tree was generated using MEGA 11. All amino acid sequences of LPMOs were acquired from the NCBI database by searching for GenBank ID. Structure-based sequence alignment was achieved by using the SALIGN module of MODELLER within PyMod 3.0 [13]. Alignment visualization was created using ESPript 3.0 [14] online analysis tool of (http://espript.ibcp.fr/ESPript/ESPript/ (accessed on 23 June 2025)).

The *Sd*LPMO10A enzyme’s three-dimensional (3D) structure was constructed using the AlphaFold 3 online platform (https://golgi.sandbox.google.com/ (accessed on 23 June 2025)). Subsequently, the obtained protein model was visualized using PyMOL 3.0 (http://www.pymol.org (accessed on 23 June 2025)), specifically labeling the active site and chelated copper ion within *Sd*LPMO 10A.

### 2.4. Effects of Temperature and pH on SdLPMO10 Activity and Stability

The method of Breslmayr et al. [15] was used, and enzyme activity was determined using 2,6-dimethylphenol (2,6-DMP) and H_2_O_2_ as co-substrates and quantified by measuring the change in absorbance at 469 nm after 5 min of incubation. The unit of enzyme activity was defined as the quantity of recombinant *Sd*LPMO10A that produced 1 μmol of oxidized product (with ε_469_ = 53,200 M^−1^ cm^−1^) per minute in the reaction system.

To determine the optimal temperature, the effect of varying temperatures (20–70 °C) in 20 mM Bis–Tris buffer (pH 6.0) on *Sd*LPMO10A activity was examined, indicating the maximal activity as 100%. To ascertain temperature stability, *Sd*LPMO10A was incubated at temperatures ranging from 20 to 60 °C for durations of 2–10 h in 20 mM Bis–Tris buffer (pH 6.0).

To determine the optimal pH value of *Sd*LPMO10A, activity measurements across pH 6.0–10.0 were performed using the following buffers (20 mM): Bis–Tris (pH 6.0–7.0), Tris-HCl (pH 8.0–9.0), CHES-NaOH (pH 10.0). We set the maximum enzyme activity to 100%, and the enzyme activities under other conditions are expressed as relative activities. To investigate the effect of pH on the stability of *Sd*LPMO10A, the enzyme protein was incubated in buffers with different pH values (6.0–10.0) for 2–24 h, and its residual activity was then determined. The initial *Sd*LPMO10A activity without buffer treatment was considered as the 100% benchmark.

### 2.5. Chitin and Cellulose Degradation Experiments

To assess *Sd*LPMO10A’s chitin activity, a 1% (*w*/*v*) α-chitin substrate underwent incubation with 10 µM Cu^2+^-saturated *Sd*LPMO10A and 1 mM ascorbic acid in 20 mM Tris-HCl buffer (pH 8.0). Following a 48 h reaction period at 30 °C, the mixture was centrifuged at 10,000× *g* for 5 min, separating the supernatant from the substrate. A control system lacking ascorbic acid or *Sd*LPMO10A was used for comparison.

Regarding cellulose degradation, 0.5% (*w*/*v*) phosphoric acid swollen cellulose (PASC), prepared as described by Zhang et al. [16], was incubated with 10 µM Cu^2+^-saturated *Sd*LPMO10A and 1 mM ascorbic acid in 20 mM Tris-HCl buffer (pH 8.0). Following a 96 h reaction period at 30 °C and 1000 rpm, the mixture was centrifuged at 10,000× *g* for 5 min, separating the supernatant from the substrate. A control system lacking ascorbic acid and *Sd*LPMO10A was used for comparison.

### 2.6. MALDI-TOF MS Analysis

Matrix-assisted laser desorption ionization–time-of-flight mass spectrometry (MALDI-TOF MS) analysis of oxidized chitin oligosaccharides within the supernatant was carried out using the modified method from Wang et al. [12]. It involved mixing 10 µL of reaction products with 6 µL of 10 mM NaCl, along with 10 µL of 10 mg mL^−1^ 2,5-dihydroxybenzoic acid (DHB) in a solution of 50% acetonitrile and 0.1% trifluoroacetic acid. This mixture (1 µL) was spotted on a steel target, air-dried to form crystals, and analyzed using the MALDI-7090 mass spectrometer (Shimadzu, Kyoto, Japan) in reflective mode.

### 2.7. Structural Analysis of α-Chitin After SdLPMO10A Pre-Treatment

The post-enzyme reaction substrate was subjected to further analyses. The method of Soon et al. [17] was employed, and scanning electron microscopy (SEM) at an accelerated voltage of 15 kV (Phenom Pro 10102, Phenom World, Eindhoven, The Netherlands) was used to obtain photomicrographs at various magnifications. The method of Gbenebor et al. [18] was used, and Fourier transform infrared (FTIR) spectroscopy in attenuated total reflection (ATR) mode (Spectrum100, Perkin Elmer, Waltham, MA, USA) was conducted to assess alterations in functional groups across the range of 400–4000 cm^−1^ with a resolution of 4 cm^−1^ and a scanning frequency of 32 times. Additionally, an X-ray diffractometer (TD-3500, Danton Tongda Science & Technology Co., Ltd., Dandong, China) was used according to the method of Zhou et al. [19] for X-ray diffraction (XRD) analysis, with copper radiation (25 mA, 35 kV), scanning 2θ from 5° to 35° with steps of 0.02°. The crystallinity index (*CrI*; %) was compared between *CrI*_110_ and *CrI*_020_ using the following calculation formula:

*CrI*_020_ = (*I*_020_ − *I*_am_)/*I*_020_ × 100%; *CrI*_110_ = (*I*_110_ − *I*_am_)/*I*_110_ × 100%;

*I*_020_ and *I*_110_ are the maximum intensities at 10° and 22°, respectively. The intensity of amorphous diffraction (*I*_am_) was obtained at about 16°.

### 2.8. Synergy Test of SdLPMO10A and Chitinase

The reaction setup involved 0.5 U/mL of commercial chitinase (about 83 µM), with 1 U releasing 1.0 mg of *N*-acetyl-D-glucosamine (NAG) from chitin per hour in 20 mM Bis–Tris buffer (pH 6.0) at 25 °C in a 2–120 h assay, along with 5 µM Cu^2+^-saturated *Sd*LPMO10A and 1 mM ascorbic acid in 20 mM Tris-HCl buffer (pH 8.0). The molar ratio of LPMO to chitinase is approximately 1:16.5. The degradation of chitin, combinedly mediated by LPMO and chitinase, encompassed two distinct procedures: a one-pot method involving simultaneous treatment with LPMO and chitinase, and a two-step method comprising initial treatment with LPMO for 48 h followed by the addition of chitinase.

The method of Katta et al. [20] was used, and the analysis of chitin oligosaccharides in the hydrolysates was conducted using a high-performance liquid chromatography (HPLC) system (LC-20, Shimadzu, Japan) equipped with a polymer amino column Asahipak NH_2_P-50 4E (Shodex, Tokyo, Japan). The mobile phase consisted of acetonitrile/water (70/30), maintained at a column temperature of 30 °C and operated at a flow rate of 0.7 mL/min. Monosaccharide and chitin oligosaccharides were detected at 210 nm using an ultraviolet detector (UV).

### 2.9. Statistical Analysis

IBM SPSS Statistics 22 software was used for significant difference analysis, and Origin 2021 software and Figdraw 2.0 were used for mapping. All experiments were conducted in triplicate (*n* = 3), and the results are presented as mean ± standard deviation (SD); *p* < 0.05 was considered to indicate a significant difference.

## 3. Results and Discussion

### 3.1. Heterologous Expression and Purification of SdLPMO10A

**Figure 1:**
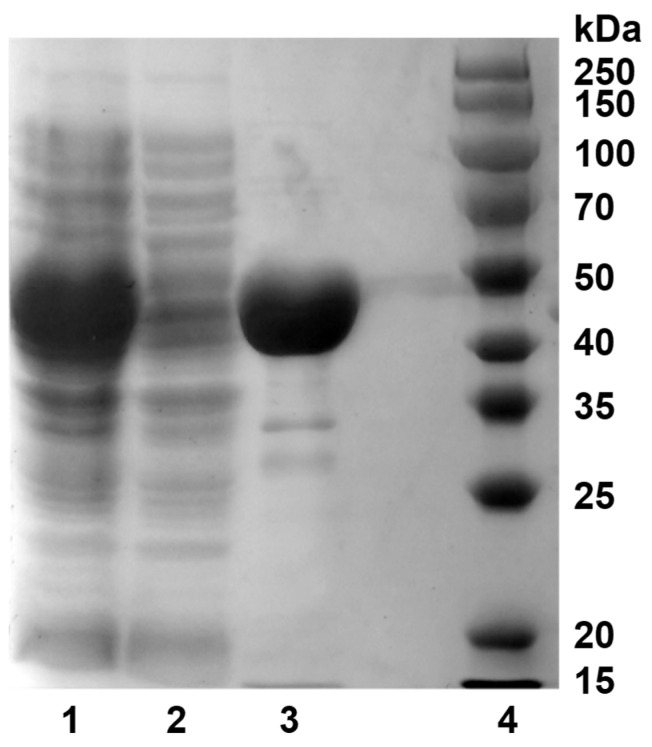
SDS-PAGE of the recombinantly produced *S. degradans* 2-40^T^ LPMO *Sd*LPMO10A. Lane 1, crude protein extract from *E. coli* periplasm; lane 2, Ni-NTA column flow-through solution; lane 3: purified *Sd*LPMO10A; lane 4: protein marker.

Successful expression of recombinant *Sd*LPMO10A in *E. coli* was confirmed. Figure 1 illustrates the results obtained from SDS-PAGE, indicating that the molecular weight (MW) of the target protein falls within the range of 40–50 kDa. This observed size is in close agreement with the predicted molecular weight of *Sd*LPMO10A (46,195.12 Da).

### 3.2. Enzyme Specificity Assay of SdLPMO10A

**Figure 2:**
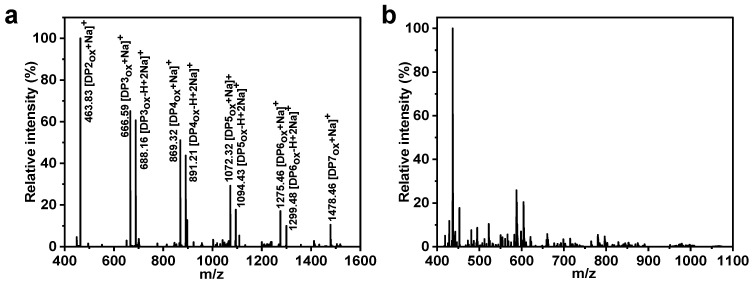
MALDI-TOF MS analysis of the products produced by treating α-chitin with *Sd*LPMO10A (**a**) and obtained in the control containing only α-chitin (**b**). The mass-to-charge ratio (*m*/*z*) and forms of each significant peak corresponding to the products are labeled above each peak. The data are as follows: DP2_ox_ GlcNAcGlcNAc1A *m*/*z* 463.83 [M + Na^+^]; DP3_ox_ (GlcNAc)_2_GlcNAc1A *m*/*z* 666.59 [M + Na^+^] and 688.16 [M-H+2Na^+^]; DP4_ox_ (GlcNAc)_3_GlcNAc1A *m*/*z* 869.32 [M+Na^+^] and 891.21 [M-H + 2Na^+^]; DP5_ox_ (GlcNAc)_4_GlcNAc1A *m*/*z* 1072.32 [M + Na^+^] and 1094.43 [M-H+2Na^+^]; DP6_ox_ (GlcNAc)_5_GlcNAc1A *m*/*z* 1275.46 [M + Na^+^] and 1299.48 [M-H + 2Na^+^]; DP7_ox_ (GlcNAc)_6_GlcNAc1A *m*/*z* 1478.46 [M + Na^+^]. Notably, the *m*/*z* difference between adjacent DP was at 203.

Based on phylogenetic analysis (Figure S1) of other known AA10 family LPMOs, *Sd*LPMO10A was identified as a prospective cellulose-active LPMO. As shown in Figure S2, the cellulose activity of *Sd*LPMO10A by C1 oxidation was confirmed though MALDI-TOF MS. The oxidative activity of *Sd*LPMO10A on chitin was also verified, as depicted in Figure 2. The spectrogram illustrates signals corresponding to oxidized chitin oligosaccharides spanning different degrees of polymerization (DP 2–7), indicative of *Sd*LPMO10A’s oxidative activity on α-chitin. Moreover, the characteristic C1-oxidized products of LPMOs, specifically aldonic acids, were observed, presenting unique MS signals attributed to the presence of sodium ions. Predominantly, both the single sodium adduct and the double sodium adduct forms of aldonic acid were evident. Signals indicating the formation of C4-oxidized products were not detected. This emphasizes the confirmed C1-oxidative regioselectivity of *Sd*LPMO10A. Furthermore, in the negative control reaction lacking *Sd*LPMO10A or ascorbic acid, no discernible quantity of oxidized chitin oligosaccharides was generated.

In summary, we first characterized an AA10-LPMO from *Saccharophagus degradans* 2-40^T^ with substrate diversity. However, considering the limited number of characterizations of chitin-active LPMOs compared to cellulose-active LPMOs presently available, we mainly explored its application in chitin degradation. It is speculated that residues more remote from copper may indirectly affect the active site, leading to differences in substrate specificity, such as the aromatic residues and cavity in the binding surface of LPMO [21]. The key factors determining substrate specificity are currently unknown, and further elucidation of specific mechanisms is required in the future.

### 3.3. Characterization of the Properties of SdLPMO10A

**Figure 3:**
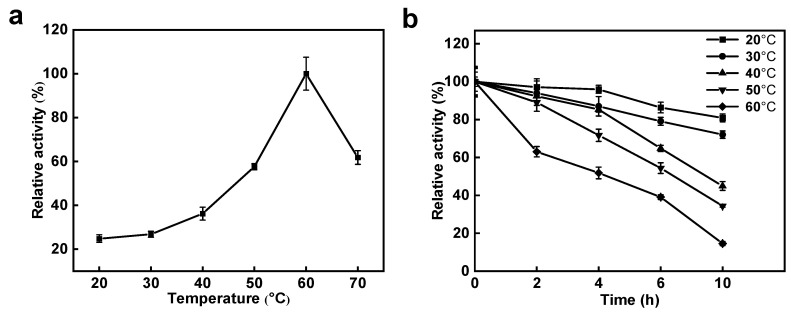
Characterization of enzymatic properties of *Sd*LPMO10A. Effect of different temperatures (20–70 °C) on activity (**a**) and stability (**b**) of *Sd*LPMO10A.

The impact of varying temperatures on *Sd*LPMO10A activity is depicted in Figure 3a. The catalytic efficacy of *Sd*LPMO10A exhibited a gradual rise with an escalating temperature, reaching highest oxidation activity at 60 °C. Remarkably, over 60% relative activity was observed even at the high temperature of 70 °C. Assessing *Sd*LPMO10A’s thermal stability across different temperatures was pivotal, as shown in Figure 3b. Notably, after 10 h of incubation at 20 °C and 30 °C, *Sd*LPMO10A retained 80.91% and 72.06% of its activity, respectively, and preserved 64.78% of its activity after 6 h of incubation at 40 °C. However, the residual activity of *Sd*LPMO10A rapidly decreased at 60 °C and was only 14.61% after 10 h of incubation. The results suggest that *Sd*LPMO10A is temperature-sensitive and cannot tolerate high temperatures above 40 °C.

Enzyme thermostability is modulated by multiple factors. Ionic liquids (ILs) enhance LPMO stability at high temperatures (e.g., *Sc*LPMO10B/C [22]) by providing a stable microenvironment, leveraging their low volatility, thermal stability, and solubility. Similarly, DESs (e.g., ChCl: glycerol/urea [23]) boost LPMO thermostability via mechanisms akin to those of ILs, forming protective hydration layers that regulate ionic strength/pH and mitigate thermal denaturation. Furthermore, Sunna [24] and Couturier et al. [25] indicated that the CBM may enhance enzyme thermal stability. Carbohydrate-binding modules (CBMs) can stabilize enzymes (e.g., *Nc*LPMO9C) through substrate binding, yet their effects are context-dependent: linker truncation impairs *Nc*LPMO9C stability [26], while *Ta*AA9A’s CBM reduces thermostability by ~20 °C [27]. This structural/substrate-specific influence necessitates further mechanistic studies for industrial enzyme optimization. Therefore, further investigation is needed to elucidate the mechanism behind the CBM domain’s influence on *Sd*LPMO10A’s thermal stability.

**Figure 4:**
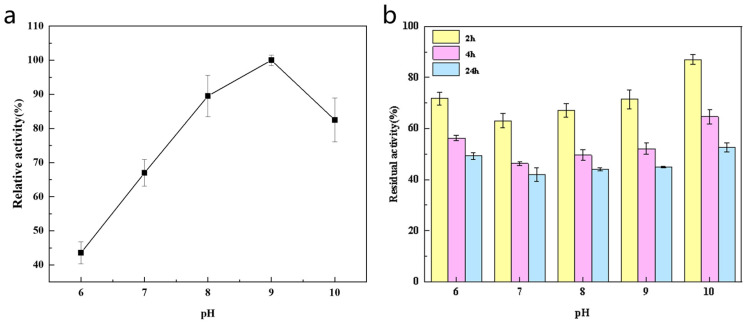
The effect of pH on the activity (**a**) and stability (**b**) of *Sd*LPMO10A.

The influence of pH on the activity of *Sd*LPMO10A is shown in Figure 4a. Within the pH range of 6.0 to 9.0, the activity of *Sd*LPMO10A continuously increased with the increase in pH. On the contrary, within the pH range of 9.0 to 10.0, the activity gradually decreased as the pH increased. The best 2, 6-DMP oxidation activity was observed at pH 9.0. However, at pH 10.0, *Sd*LPMO10A still retained more than 80% of its activity, indicating that it maintains high activity under alkaline pH conditions. The optimal pH value of *Sd*LPMO10A is higher than that of *Bat*LPMO10 from Bacillus subtilis and of most reported AA9 LPMOs [28,29]. It should be noted that the optimal pH value for LPMO activity depends on reaction conditions and substrate [29]. The influence of pH on the stability of *Sd*LPMO10A is shown in Figure 4b. Under the conditions of pH 6.0 to 10.0, the residual enzyme activity of *Sd*LPMO10A generally decreased, being 49.3%, 41.9%, 44.0%, 44.9% and 52.5% of the initial activity, at the different pH values examined. Furthermore, as the pH value increased, the stability of the enzyme significantly increased, indicating that *Sd*LPMO10A is stable under strongly alkaline conditions and can tolerate a strongly alkaline environment for a certain period of time. Some carbohydrate hydrolases also exhibit a similar behavior [30].

### 3.4. Structural Insights on SdLPMO10A

The core breakthrough of AlphaFold 3 lies in the adoption of a diffusion model (an AI technology similar to image generation) to achieve its “joint” prediction framework. Unlike AlphaFold 2, which only predicts protein structures, AlphaFold 3 can simultaneously predict the structure and the interactions of complete biomolecular complexes composed of proteins, nucleic acids (DNA/RNA), and small-molecule ligands (including key metal ions) and their chemical modifications [31]. By taking advantage of this powerful joint modeling capability, we successfully constructed a three-dimensional structural model of *Sd*LPMO10A using the AlphaFold 3 platform.

**Figure 5:**
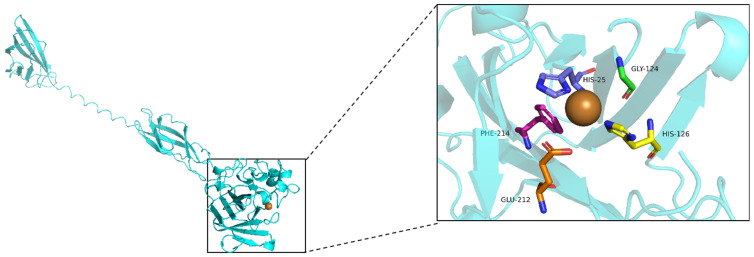
The active site structure in *Sd*LPMO10A. Three-dimensional structure and active site of *Sd*LPMO10A, with the α-helices represented by blue curls, and the β-sheets indicated by blue arrows. The copper ion is depicted as a red sphere, while the coordinating histidine side chains (His25 and His126) are denoted by purple and yellow rod shapes, respectively. Gly124 and Glu212 are denoted by green and orange rod shapes, respectively.

The model of *Sd*LPMO10A revealed a conserved immunoglobulin-like β-sheet core along with a histidine brace (comprising the *N*-terminal histidine His25 and His126) (Figure 5). Structurally, *Sd*LPMO10A comprises two β-sheet formed by four antiparallel β-strands each and two α-helices, housing two histidine residues forming the LPMO active sites within a β-sandwich structure, resembling other reported AA10 LPMOs. Most of the putative AA10 LPMOs derived from bacteria show a conserved alanine residue in the second coordination sphere of the active site Cu (II) ion, but it was found that alanine residues can be replaced by multiple amino acids [32]. Interestingly, multiple sequence alignment revealed that no conserved alanine or isoleucine residues are present within the second coordination sphere of *Sd*LPMO10A, but rather glycine residues were identified. This result is consistent with findings obtained for *Tt*AA10A, *Cj*AA10B, and *Hc*AA10A, which belong to the same subclade (see Figure S3 for details). The substitution of the second-coordination-sphere alanine residue by the less bulky glycine (Gly124) may cause a slightly more axial coordination geometry closer to the one typical of AA9 LPMOs and cellulose-active AA10 LPMOs [32,33]. The other two conserved second-coordination-sphere residues are phenylalanine (Phe214) and glutamine/glutamate (Glu212) residues, which often coexist in AA10 LPMOs. It is noteworthy that the second-coordination-sphere glutamic acid, a structurally conserved “gatekeeper” residue, occupies different positions in the LPMO sequence (E60 in *Sm*AA10A, E212 in *Sd*LPMO10A), depending on the subclade [34], and is responsible for positioning and constraining H_2_O_2_ and the hydroxyl radical [35].

### 3.5. Structure of α-Chitins After SdLPMO10A Pre-Treatment

**Figure 6:**
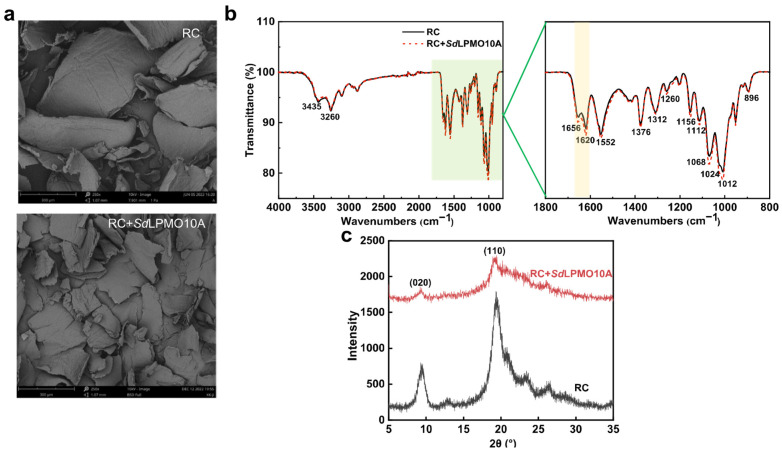
Structural analysis of α-chitin after *Sd*LPMO10A pre-treatment. (**a**) SEM images of *Sd*LPMO10A-treated (lower) and -untreated raw chitin (RC, upper). (**b**) FTIR analysis of *Sd*LPMO10A-untreated (black line) and -treated chitin (red dashed line). The characteristic absorption peaks are principally at 3435 cm^−1^ (caused by the O-H stretching vibration), 3260 cm^−1^ (N-H stretching vibration), 2961–2840 cm^−1^ (C-H stretching vibration), 1160–1010 cm^−1^ (C-O stretching vibration), 1656 cm^−1^ and 1620 cm^−1^ (C=O, amide I band), 1552 cm^−1^ (N-H, amide II band), 1312 cm^−1^ (C-N, amide III band), and 896 cm^−1^ (stretching vibration of the *β*-1,4 glycosidic bond) [37]. (**c**) XRD patterns of *Sd*LPMO10A-oxidized chitin (red line) and the non-oxidized control RC (black line).

The SEM images obtained offer a visual insight into the surface morphology of α-chitin before and after treatment with *Sd*LPMO10A. Figure 6a illustrates a noticeable reduction in the size of α-chitin after *Sd*LPMO10A treatment compared to that in the untreated sample. This reduction aligns with findings demonstrating *Sm*AA10A’s ability to independently decompose large β-chitin crystals [3]. Eibinger et al. [36] showed that LPMO oxidation can induce surface puncturing of resilient polymers, leading to a decrease in particle size.

The FTIR spectra of *Sd*LPMO10A-treated and -untreated chitin show identical peak positions and shapes in Figure 6b, suggesting that *Sd*LPMO10A had no significant impact on the chemical structure of chitin. However, the treated α-chitin displayed weaker and smoother absorption peaks at 3435 cm^−1^, indicative of hydrogen bonding interactions [37], implying that *Sd*LPMO10A may disrupt the hydrogen bonding network. Significantly, an increase in peak intensity at 1656 and 1620 cm^−1^ (C=O, amide I) was observed, indicating oxidative modification of the chitin surface [38]. In addition, compared with RC, *Sd*LPMO10A treatment increased the absorption at 1700–1000 cm^−1^, which is in accord with the result obtained for *Sm*AA10A-treated α-chitin [39].

Examining the XRD pattern of α-chitin treated with *Sd*LPMO10A (Figure 6c), the main diffraction peak remained around 19.2° for 2θ, indicating the presence of the typical chitin crystal structure observed in the untreated sample [40]. However, smaller diffraction peaks at 9.4°, 23.4°, and 26.3° indicated a disruption in the crystallization zone during the treatment process, resulting in *CrI*_020_ and *CrI*_110_ values of 53.4% and 75.2%, respectively. This decrease in chitin crystallinity may stem from bifurcated hydrogen bonds. The critical hydrogen bonds between the C=O group and the N-H group within the same chain, along with the O-H group of adjacent sugars, contribute significantly to chitin’s crystallinity [41]. The *Sd*LPMO10A treatment appeared to weaken this hydrogen bond network, consistent with the FTIR analysis. Consequently, the degree of order and crystallinity diminished, inducing a tendency towards the production of amorphous chitin, potentially enhancing chitinase accessibility and facilitating the production of chitin oligosaccharides.

### 3.6. Synergistic Effect of SdLPMO10A and Chitinase

**Figure 7:**
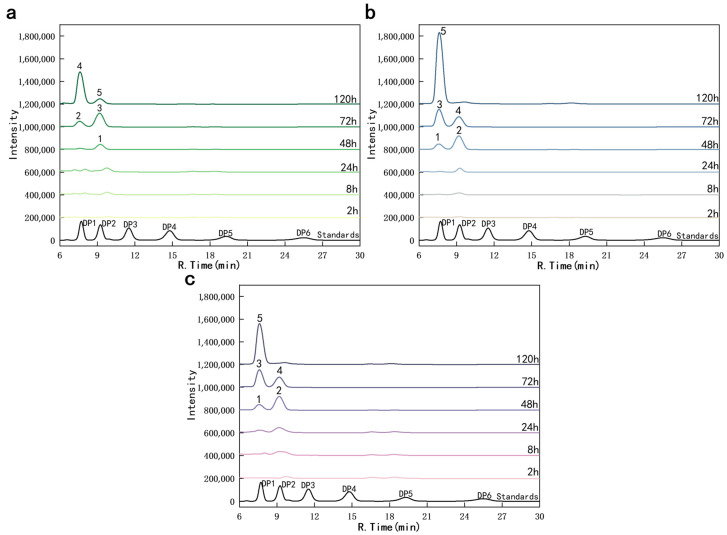
Qualitative and quantitative analysis of enzymatic hydrolysis products. HPLC analysis: (**a**) one-pot reaction using only commercial chitinase, (**b**) simultaneous addition of *Sd*LPMO10A and chitinase, and (**c**) two-step reaction: before the reaction, α-chitin is pretreated with *Sd*LPMO10A for 48 h, and then chitinase is added for co-treatment for 0–120 h. The peak areas corresponding to the numbers indicated in the figure are provided in Table 1.

The qualitative analysis of the enzymatic hydrolysis products using HPLC (Figure 7) demonstrated that NAG and the disaccharide (NAG)_2_ were the primary products of the reaction. Over time, the generated (NAG)_2_ ultimately transformed into NAG. The yield of (NAG)_2_ in the *Sd*LPMO10A- and chitinase-treated sample peaked after 48 h (Figure 7b), whereas it peaked after 72 h when using chitinase alone (Figure 7a). After 120 h, when chitinase alone was used, NAG and (NAG)_2_ were still present, while the synergistic degradation almost completely converted chitin into NAG, indicating that *Sd*LPMO10A accelerated the reaction process. Furthermore, the peak area of the synergistic degradation was notably larger than that of the reaction with chitinase alone, with the one-pot degradation method showing the most significant difference (Figure 7c and Table 1). Although low concentrations of Cu^2+^ (10 µM) and ascorbic acid (1 mM) were used, which are unlikely to significantly inhibit chitinase based on literature reports of their effects at higher levels, this represents a potential limitation that could be explored in future optimizations [42,43].

However, it is noteworthy that the commercial chitinase used might be more suitable for amorphous chitin, while the *Serratia marcescens* enzyme (*Sm*AA10A) seems more adept at handling highly crystalline substrates [39]. Hence, customizing a blend of enzymes based on substrate characteristics could be pivotal in achieving the maximum product yield. This indicates the importance of tailoring mixed enzyme combinations to the substrate’s properties for optimal efficiency in chitin degradation. From a food technology standpoint, *Sd*LPMO10A offers the possibility to create a mild, enzyme-based platform for biorefining chitin waste from the food industry into high-value chitooligosaccharides, potentially reducing the environmental impacts of shellfish processing and enabling the formulation of novel functional foods with prebiotic and health-promoting properties. Future optimization could integrate this LPMO into industrial food enzyme cocktails for scalable production.

## 4. Conclusions

In this study, we characterized *Sd*LPMO10A, an AA10 family LPMO from *Saccharophagus degradans* 2-40^T^, demonstrating its substrate diversity, with activity on both chitin and cellulose, though we focused on its chitin-degrading potential. Enzymatic assays revealed optimal pH of 9.0 and temperature of 60 °C, with notable stability under alkaline conditions (residual activity >40% after 24 h at pH 9.0–10.0). Structural analyses (SEM, FTIR, and XRD) showed that *Sd*LPMO10A reduces chitin particle size, disrupts hydrogen bond networks, and lowers crystallinity (*CrI*_020_: 53.4%; *CrI*_110_: 75.2%), thereby enhancing accessibility for chitinase. In synergy experiments, *Sd*LPMO10A increased chitooligosaccharide yields, with the one-pot method outperforming the two-step approach by accelerating hydrolysis and yielding primarily N-acetyl-D-glucosamine after 120 h. These results provide insights into *Sd*LPMO10A’s mechanism of action, including its C1-oxidative regioselectivity and unique second coordination sphere (e.g., Gly124 substitution), and suggest its utility in enzymatic chitin degradation under mild conditions. Potential limitations include the untested effects of copper and ascorbic acid on chitinase activity, though the literature suggests minimal inhibition at our concentrations. Additionally, optimizing the assay conditions—such as pH, enzyme molar ratios, and reductant levels—alongside the development of more efficient chitinases tailored to crystalline substrates, represents a key avenue for enhancing LPMO-GH synergies in future studies. However, limitations in the current detection methods for oxidized products constrain further progress in LPMO research. Future work could refine analytical techniques to quantify the described synergy more precisely and explore industrial-scale applications in biomass conversion.

## Figures and Tables

**Table 1:** The area of each peak in Figure 7 after 120 h.

Peak	Compound	Peak Area (×10^4^) (mV·min)	FWHM (min)
a-4	NAG	17.4369	0.5869
a-5	(NAG)_2_	3.6131	0.7215
b-5	NAG	40.0459	0.6127
c-5	NAG	22.9042	0.6066

The peak areas at 48–72 h in Figure 7 are reported in the Supplementary Materials (Table S1). The half-peak width of FWHM should be between 0.1 and 1.0 min.

## Data Availability

The original contributions presented in the study are included in the article, further inquiries can be directed to the corresponding author.

## Supplementary Materials

The following supporting information can be downloaded at: https://www.mdpi.com/article/10.3390/foods14162839/s1, Figure S1: Maximum-likelihood phylogenetic tree of *Sd*LPMO10A and currently characterized AA10 LPMO catalytic modules from Li et al., 2021 [44]. The LPMO characterized in this study is marked with a red five-pointed star, and LPMOs used to for further sequence alignment are marked with black solid circles; Figure S2: Verification of cellulose activity of *Sd*LPMO10A by MALDI-TOF MS. The reaction between *Sd*LPMO10A and PASC in the presence of ascorbic acid (a), and the reaction only with PASC (b). Soluble products were never observed in PASC (results not shown in a). The mass-to-charge ratio (*m*/*z*) and forms of each significant peak corresponding to the product are labeled above. Apart from dehydrated oligosaccharides produced by phosphoric acid treatment (DP2, *m*/*z* 347.42), only the products in an aldonic acid form were observed, the data are as follows: DP2ox GlcGlc1A *m*/*z* 381.62 [M + Na+]; DP3ox (Glc)2Glc1A *m*/*z* 543.73 [M+Na+]; DP4ox (Glc)3Glc1A m/z 705.63 [M + Na+]; DP5ox (Glc)4Glc1A *m*/*z* 867.71 [M + Na+]; DP6ox (Glc)5Glc1A *m*/*z* 1029.75 [M + Na+]. The *m*/*z* difference between adjacent DP stands at 162; Figure S3: Structure-based sequence alignment between *Sd*LPMO10A and other AA10 LPMOs (*Sm*LPMO10A, *Hc*LPMO10, *Cj*LPMO10B and *Tt*LPMO10A). The signal peptide of 24 amino acids in *Sd*LPMO10A has been removed. The two histidine residues (H25 and H126) that form the copper binding site are marked with orange six-pointed stars, while the exposed aromatic residue phenylalanine (F214) involved in substrate binding is marked with a blue triangle. The conserved alanine/isoleucine in AA10 LPMO is replaced by glycine and marked as a green diamond. The structurally conserved “gatekeeper” residues (Glu/Gln) are marked with bright blue and green borders; Table S1: The area of each peak at 48–72 h.

## Abbreviations

*N*-acetyl-D-glucosamine (GlcNAc, NAG); lytic polysaccharide monooxygenases (LPMOs); glycoside hydrolases (GHs); auxiliary activity (AA); carbohydrate-binding module (CBM); sodium dodecyl sulfate-polyacrylamide gel electrophoresis (SDS-PAGE); Luria–Bertani (LB); National Center for Biotechnology Information (NCBI); isopropyl β-D-1-thiogalactopyranoside (IPTG); 2,6-dimethylphenol (2,6-DMP); matrix-assisted laser desorption ionization–time-of-flight mass spectrometry (MALDI-TOF MS); 2,5-dihydroxybenzoic acid (DHB); RC (raw chitin); scanning electron microscopy (SEM); Fourier transform infrared (FTIR); attenuated total reflection (ATR); crystallinity index (CrI); X-ray diffraction (XRD); high-performance liquid chromatography (HPLC); molecular weight (MW); mass-to-charge ratio (*m*/*z*); degree of polymerization (DP); chitohexose (NAG)_6_.
