# Supplementary material for: The Functional Characterization of an AA10 Lytic Polysaccharide Monooxygenase from Saccharophagus degradans 2-40T for Enhanced Chitin Biodegradation
Source: Foods. 2025 Aug 16;14(16):2839. doi: 10.3390/foods14162839 (PMC12385910; doi:10.3390/foods14162839)
Supplement: Supplementary file 1 [file foods-14-02839-s001.zip › foods-3811678-supplementary.pdf]

Supporting Information

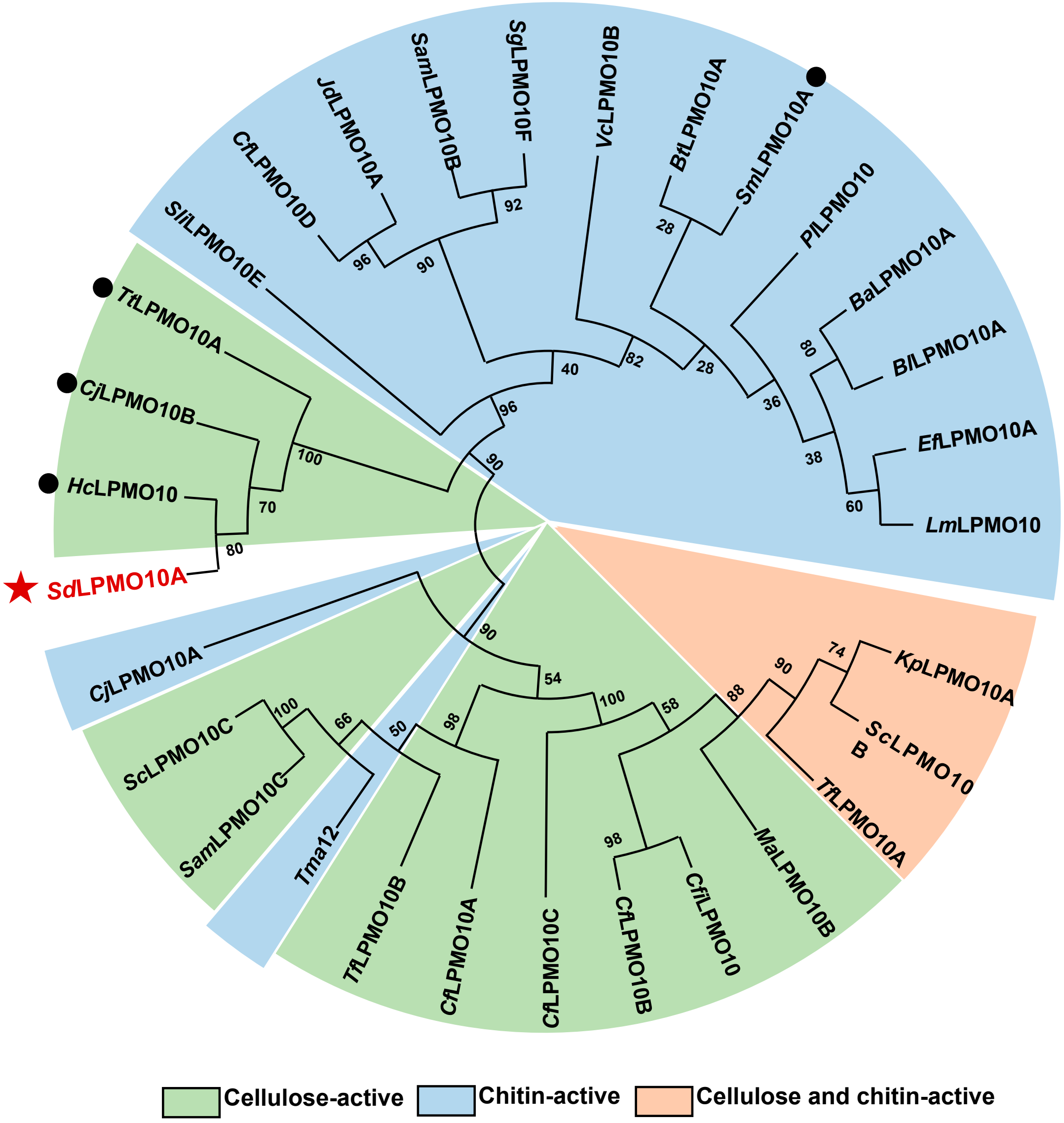

Figure S1. Maximum-likelihood phylogenetic tree of *Sd*LPMO10A and currently characterized AA10 LPMO catalytic modules from Li.et al (Li et al., 2021). The LPMO characterized in this study is marked with a red five-pointed star, and LPMOs used to for further sequence alignment are marked with black solid circles.

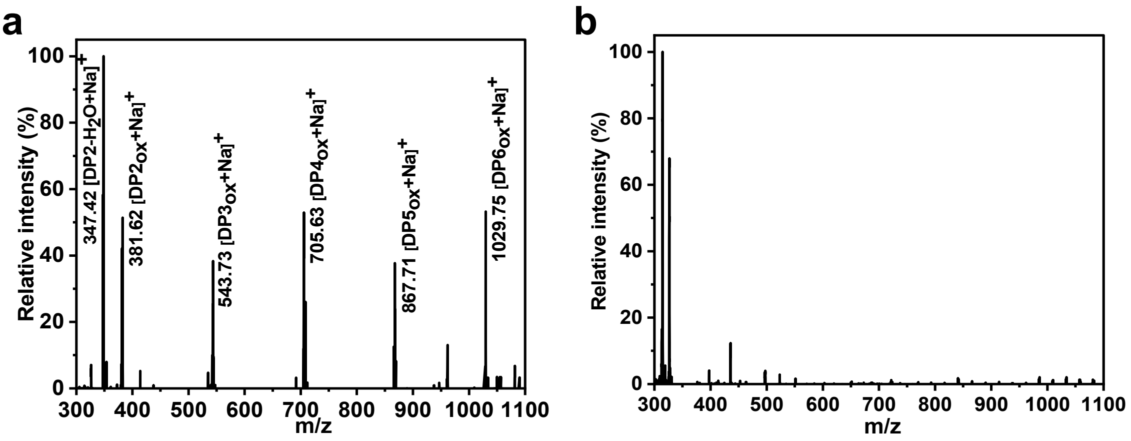

Figure S2. Verification of cellulose activity of *Sd*LPMO10A by MALDI-TOF MS. The reaction between *Sd*LPMO10A and PASC in the presence of ascorbic acid (a), and the reaction only with PASC (b). Soluble products were never observed in PASC (results not shown in a). The mass-to-charge ratio (*m*/*z*) and forms of each significant peak corresponding to the product are labeled above. Apart from dehydrated oligosaccharides produced by phosphoric acid treatment (DP2, m/z 347.42), only the products in an aldonic acid form were observed, the data are as follows: $DP2_{ox}$ GlcGlc1A *m*/*z* 381.62 [$M+Na^+$]; $DP3_{ox}$ $(Glc)_2$Glc1A *m*/*z* 543.73 [$M + Na^+$]; $DP4_{ox}$ $(Glc)_3$Glc1A *m*/*z* 705.63 [$M+Na^+$]; $DP5_{ox}$ $(Glc)_4$Glc1A *m*/*z* 867.71 [$M + Na^+$]; $DP6_{ox}$ $(Glc)_5$Glc1A *m*/*z* 1029.75 [$M + Na^+$]. The m/z difference between adjacent DP stands at 162.

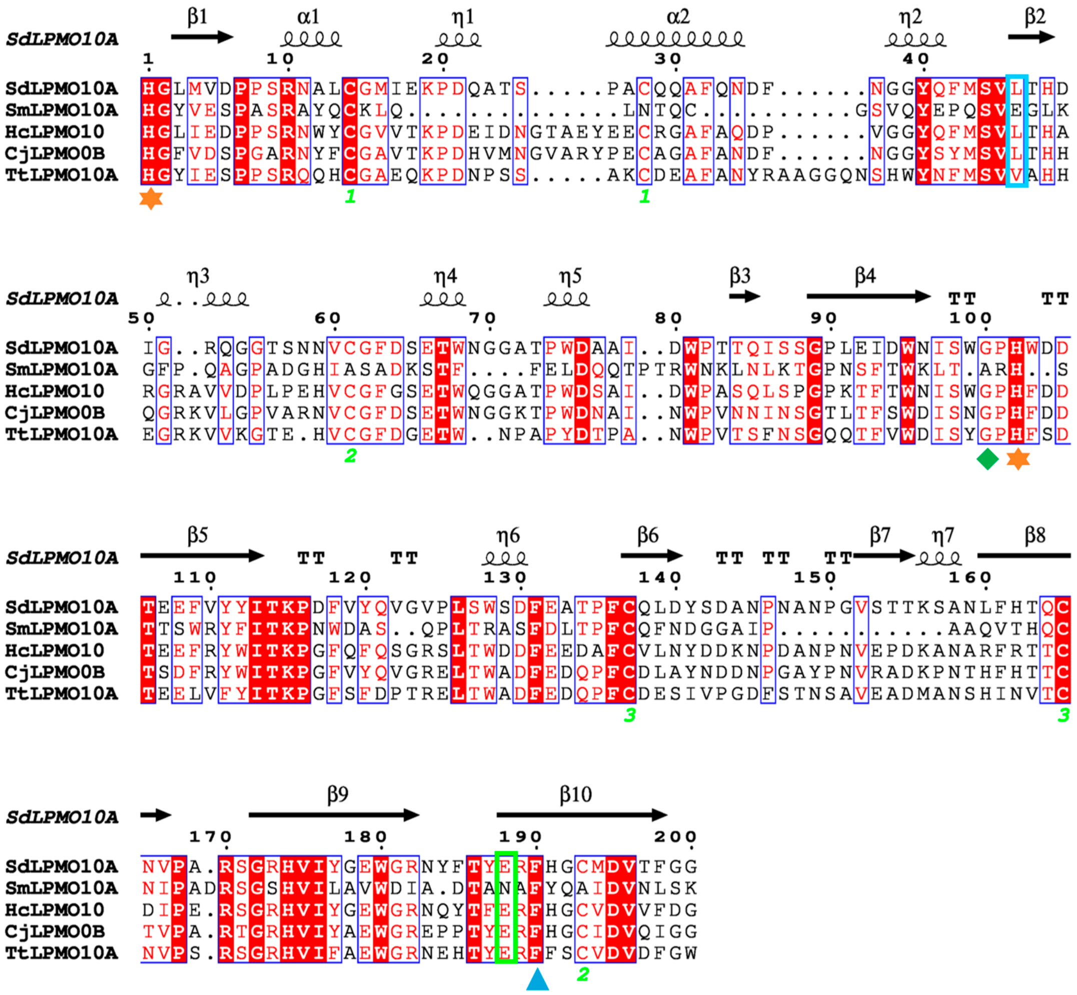

Figure S3. Structure-based sequence alignment between *Sd*LPMO10A and other AA10 LPMOs (*Sm*LPMO10A, *Hc*LPMO10, *Cj*LPMO10B and *Tt*LPMO10A). The signal peptide of 24 amino acids in *Sd*LPMO10A has been removed. The two histidine residues (H25 and H126) that form the copper binding site are marked with orange six-pointed stars, while the exposed aromatic residue phenylalanine (F214) involved in substrate binding is marked with a blue triangle. The conserved alanine/isoleucine in AA10 LPMO is replaced by glycine and marked as a green diamond. The structurally conserved "gatekeeper" residues (Glu/Gln) are marked with bright blue and green borders.

Table S1. The area of each peak at 48–72 h.

Peak	Compound	Peak Area (×10^4) (mV·min)	FWHM (min)
a-1	$(NAG)_2$	3.0064	0.6415
a-2	NAG	3.2749	0.6473
a-3	$(NAG)_2$	9.0032	0.7328
b-1	NAG	3.2484	0.6473
b-2	$(NAG)_2$	9.0420	0.7328
b-3	NAG	9.6578	0.6021
b-4	$(NAG)_2$	6.7422	0.7296
c-1	NAG	3.2905	0.6473
c-2	$(NAG)_2$	9.0282	0.7328
c-3	NAG	9.7476	0.6021
c-4	$(NAG)_2$	6.7557	0.7296
